# Targeting PAK1 Within Group I PAKs: Current Challenges and Pharmacological Perspectives

**DOI:** 10.3390/ph19101628

**Published:** 2026-10-14

**Authors:** George Nicolae Daniel Ion, Cosmina Gabriela Duta-Bratu, Dragos Paul Mihai, George Mihai Nitulescu

**Affiliations:** Faculty of Pharmacy, “Carol Davila” University of Medicine and Pharmacy, Traian Vuia 6, 020956 Bucharest, Romania; george-nicolae-daniel.ion@umfcd.ro (G.N.D.I.); cosmina-gabriela.bratu@drd.umfcd.ro (C.G.D.-B.); george.nitulescu@umfcd.ro (G.M.N.)

**Keywords:** PAK1, p21-activated kinase, kinase inhibitors, isoform selectivity, allosteric inhibition, targeted protein degradation, PROTAC, drug resistance, kinase pharmacology, cancer therapy

## Abstract

The p21-activated kinase 1 (PAK1) has been investigated as a therapeutic target for more than two decades, particularly in cancer, where it contributes to cytoskeletal regulation, proliferation, survival, migration and therapy resistance. Despite the development of numerous potent inhibitors, selective pharmacological modulation of PAK1 remains difficult because of the high similarity of the group I PAK catalytic domains, especially PAK1 and PAK2, together with differences in conformational state, cellular localization and kinase-independent functions. This review examines PAK1 within the broader PAK kinase family and critically evaluates the principal pharmacological strategies developed to modulate its activity, including ATP-competitive and allosteric inhibition, targeted degradation, polypharmacology, combination therapies and indirect approaches. Recent structural studies indicate that local conformational differences between PAK1 and PAK2 can be exploited for improved isoform discrimination, while PAK1-selective degraders provide an alternative means of affecting both catalytic and non-catalytic functions. A snapshot analysis of current ChEMBL bioactivity data further shows that, although numerous compounds display high reported potency against PAK1, matched quantitative information for PAK2 and PAK3 is available for only a limited subset, leaving isoform selectivity unresolved for most compounds. Recent findings also position PAK1 within adaptive and treatment-resistance signaling rather than as a universal oncogenic dependency. Future progress will therefore depend not only on improving compound potency, but on integrating isoform profiling, cellular target engagement, structural information and disease-specific biomarkers to identify the settings in which PAK1 modulation can provide a meaningful therapeutic advantage.

## 1. Introduction

The p21-activated kinases (PAKs) are a family of evolutionarily conserved serine/threonine kinases functioning as downstream effectors of the Rho-family GTPases, specifically Cdc42 and Rac1. Positioned at the interface between GTPase activation and downstream signaling, PAKs regulate cytoskeletal remodeling, cell polarity, proliferation, survival and transcriptional responses [1,2]. Under physiological conditions, these functions contribute to coordinating actin-microtubule dynamics and cell–cell and cell–matrix interactions. In pathological settings, the same signaling outputs support cellular invasion, altered metabolism, adaptation to stress and resistance to therapy [2,3].

Based on their sequence homology, domain organization and regulatory mechanisms, the six human PAK isoforms (PAK1–PAK6) are classified into two groups: group I (PAK1–PAK3) and group II (PAK4–PAK6). Although the two groups share a conserved kinase domain and several overlapping biological outputs, their modes of activation, tissue distribution, and interaction partners as well as physiological functions differ considerably [1,2]. These differences are directly relevant to the drug discovery field, as pharmacological inhibition of one isoform may affect related family members with distinct, and in some cases, essential physiological roles.

Among the PAK family members, PAK1 has received sustained attention since its discovery as a therapeutic target because it links Cdc42- and Rac1-dependent signaling to cytoskeletal organization, cell migration, proliferation, survival and treatment resistance in several disease contexts, particularly in cancer [3,4]. However, PAK1 is the isoform that most clearly illustrates one of the major challenges encountered in PAK-directed drug discovery, that is achieving selectivity over the other PAK family members, as its catalytic domain shows high similarity to those of PAK2 and PAK3, and its activity depends on conformational state and subcellular localization [1,4]. Moreover, some of its biological functions may not require catalytic activity [4,5]. Consequently, strong inhibition in biochemical assays does not necessarily result in selective or durable pathway modulation in cells or in vivo [4].

The present critical review examines PAK1 within the broader context of the PAK kinase family, focusing on pharmacological strategies used in recent years to modulate its activity. Recent findings are considered separately to distinguish established concepts from developments reported during the last several years, while limitations of the available pharmacological evidence and unresolved questions affecting clinical translation are critically assessed throughout the paper.

## 2. PAK1 Within the PAK Kinase Family

### 2.1. Classification and Regulation of PAK Kinases

Although all PAK isoforms share a conserved catalytic domain and a related overall architecture, the two groups differ significantly in the mechanisms controlling kinase activity. Group I PAKs contain an autoinhibitory domain that partially overlaps the CRIB motif—an N-terminal Cdc42/Rac-interactive motif, also referred to as the p21-binding domain.

PAK1 was initially described as an inactive trans-inhibited dimer, in which the autoinhibitory region of a molecule interacts with the catalytic domain of the other [6,7]. This classical model has subsequently been questioned by solution-state studies, while recent structural and biophysical analyses of full-length PAK2 support a monomeric, intramolecularly autoinhibited state and suggest that the regulatory model of group I PAKs may require revision [8]. Binding of GTP-loaded Cdc42 or Rac1 to the CRIB motif disrupts autoinhibition and promotes conformational changes and autophosphorylation events required for full catalytic activation [6,7]. Phosphorylation of residue Thr423 within the activation loop is particularly important for stabilization of the active conformation of PAK1 [9], while CK2-dependent phosphorylation at additional regulatory sites, such as Ser223, can further modulate kinase activation [10]. The activity of group I PAKs is also influenced by membrane recruitment, phosphoinositides and interactions with adaptor proteins such as NCK, GRB2 and PIX [2,4].

**Figure 1:**
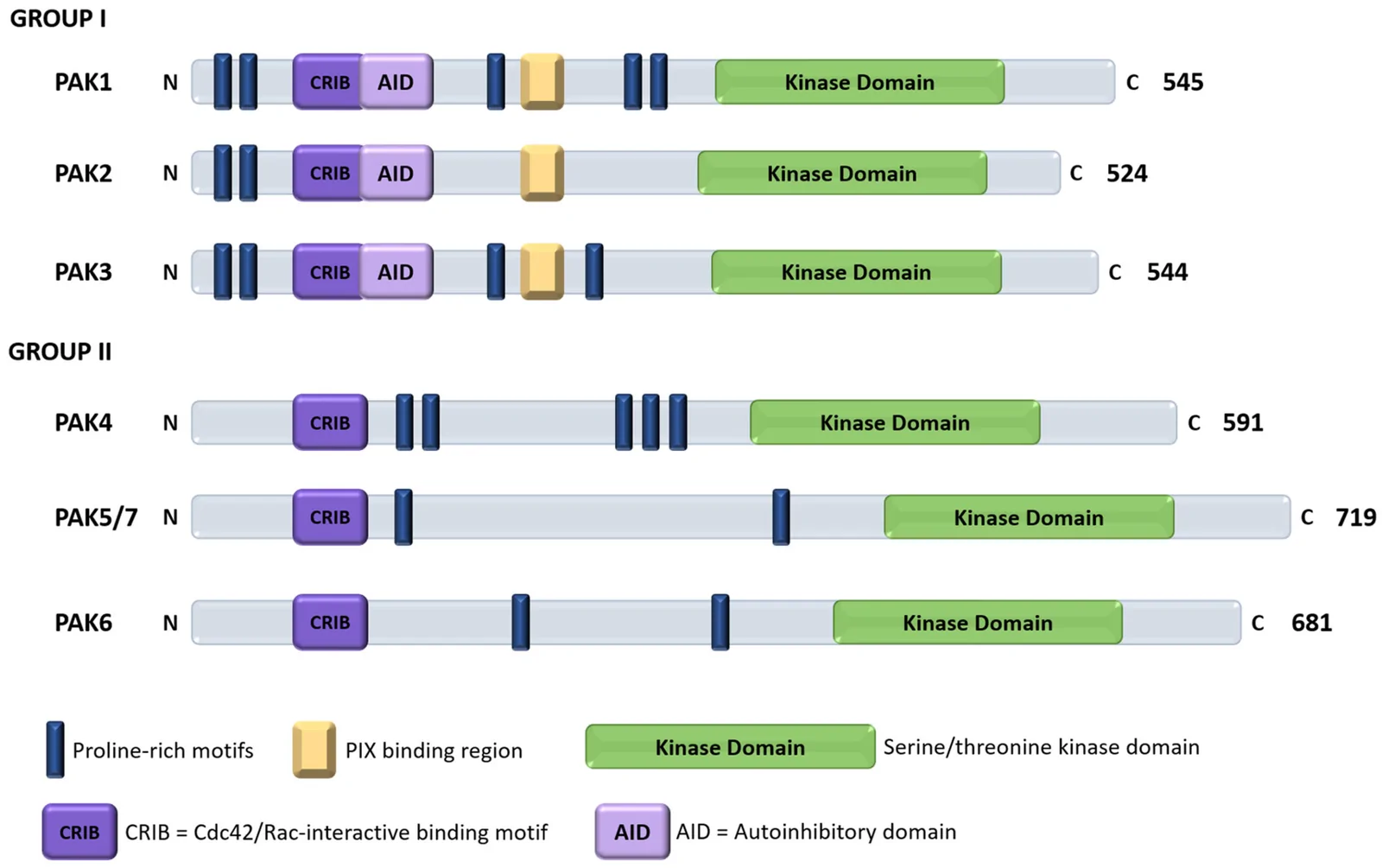
Schematic domain organization of the six human PAK isoforms. Group I PAKs (PAK1–PAK3) contain an N-terminal Cdc42/Rac-interactive binding (CRIB) motif that partially overlaps the autoinhibitory domain (AID), together with proline-rich interaction motifs and a C-terminal serine/threonine kinase domain. Group II PAKs (PAK4–PAK6) retain a CRIB motif but lack the classical group I AID and differ substantially in the organization of their regulatory regions. Protein lengths are given as numbers of amino acids. Domain positions are shown schematically and are not drawn to exact residue scale.

In contrast, group II PAKs lack the group I autoinhibitory domain and are not activated through the same GTPase-dependent release of trans-autoinhibition. Binding to Cdc42 or Rac appears to regulate their localization and protein interactions more prominently than their intrinsic catalytic activity [1,11]. For PAK4, an internal pseudosubstrate sequence restricts substrate access to the kinase domain, and a related mechanism has also been demonstrated for PAK6, whereas PAK5 regulation remains less clearly defined [11,12]. The principal differences between group I and group II PAKs, in terms of domain organization, are illustrated in Figure 1.

As the ATP-binding sites remain relatively conserved throughout the PAK family, these differences in regulatory mechanisms and conformational states surrounding the catalytic domains are directly relevant to pharmacological modulation. As a result, compounds directed toward the ATP-binding pocket frequently show activity against several PAK isoforms, while allosteric ligands exploiting group-specific regulatory conformation may achieve greater discrimination. At the same time, the difference in regulation between the two groups also signifies that an inhibitory mechanism developed for PAK1 cannot necessarily be transferred to group II PAKs.

### 2.2. PAK1 Structure and Activation

Human PAK1 is a 545 amino-acid multidomain protein composed of an N-terminal regulatory region and a C-terminal serine/threonine kinase domain. The regulatory region contains the overlapping CRIB and autoinhibitory domains, several proline-rich motifs involved in interactions with SH3-domain-containing proteins, and a basic region that contributes to membrane phosphoinositide binding [2,4]. The catalytic domain adopts the characteristic two-lobed protein kinase architecture, with the ATP-binding cleft located between the smaller N-terminal lobe and the larger C-terminal lobe [4,13].

**Figure 2:**
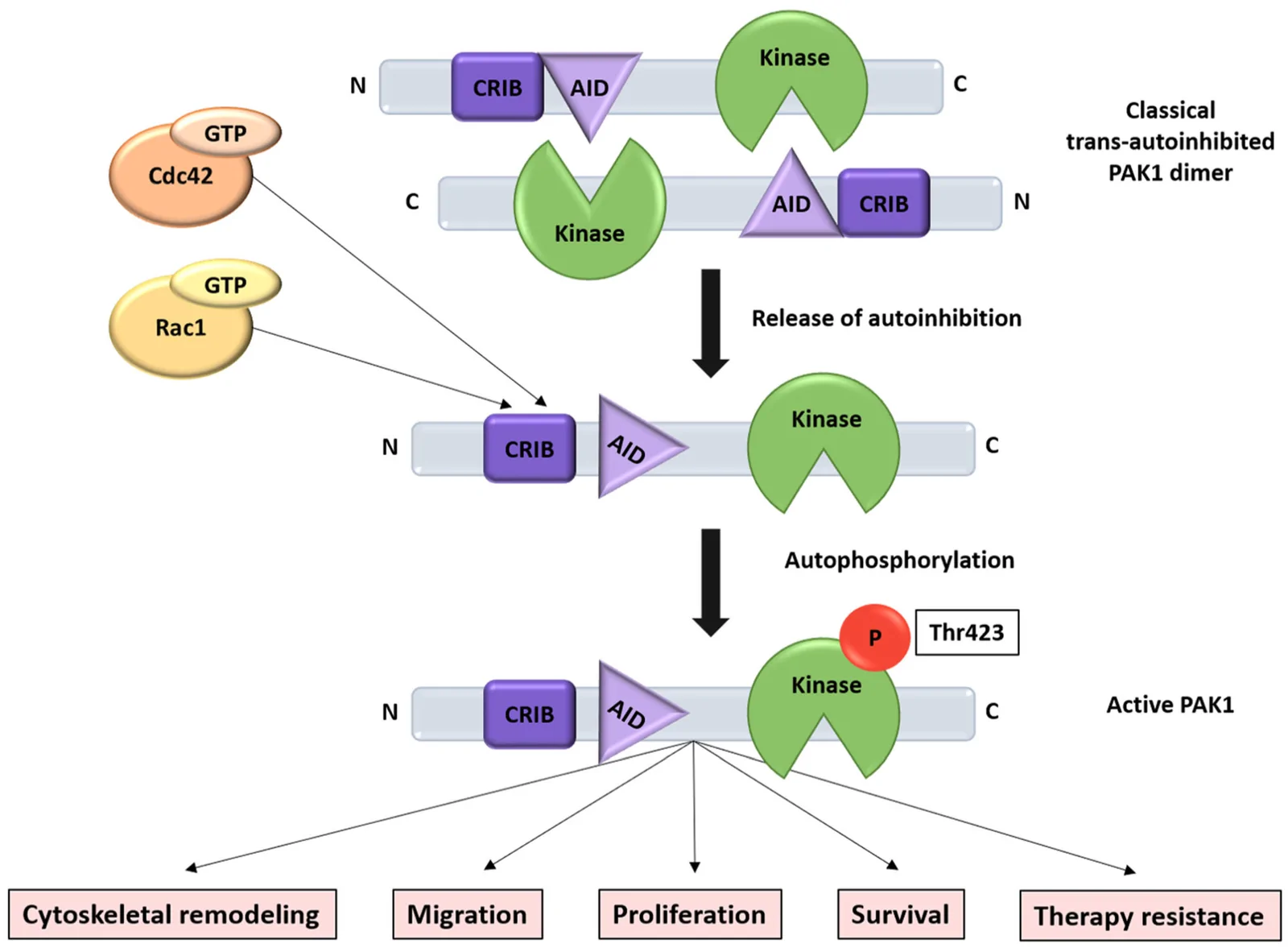
Schematic representation of PAK1 activation and major downstream cellular effects. In the classical model of group I PAK activation, GTP-bound Cdc42 or Rac1 binds the CRIB region and disrupts AID-mediated autoinhibition, permitting conformational rearrangement and autophosphorylation of Thr423 within the activation loop. Phosphorylation stabilizes the catalytically active state, which contributes to cytoskeletal remodeling, migration, proliferation, survival and therapy resistance through multiple downstream signaling networks. The classical trans-autoinhibited dimer is shown schematically, while recent full-length solution studies also support a monomeric, cis-autoinhibited configuration of PAK1.

Within the catalytic domain, the glycine-rich loop contributes to ATP positioning, while the hinge region forms hydrogen-bond interactions with the adenine moiety. The catalytic loop, activation segment and αC helix further determine whether the kinase is arranged in a catalytically competent conformation. The release of the autoinhibitory domain permits rearrangement of these structural elements, followed by phosphorylation within the activation loop and stabilization of the active kinase [6,9,13]. Structural studies of the isolated PAK1 catalytic domain suggest that release from autoinhibition may already produce an active-like intermediate, while phosphorylation of Thr423 further stabilizes the fully active state rather than acting as the only switch controlling catalytic competence [13,14]. The main steps involved in PAK1 activation and the major cellular processes associated with activated PAK1 signaling are summarized in Figure 2.

These structural transitions are important for inhibitor binding because the PAK1 kinase domain is conformationally flexible and can accommodate chemically diverse ATP-competitive ligands. However, the main residues forming the ATP-binding site are strongly conserved among group I PAKs, as the three isoforms display approximately 93–95% similarity within their catalytic domains. Therefore, conventional hinge-binding interactions provide limited opportunity for isoform discrimination, which explains the reduced selectivity for many PAK1 inhibitors that also inhibit PAK2 and, to a variable extent, PAK3 [1,4].

Nevertheless, the catalytic domains of PAK1 and PAK2 are not entirely interchangeable. Comparison of PAK1- and PAK2-inhibitor complexes revealed local differences in the conformation and flexibility of the glycine-rich loop and adjacent regions of the ATP-binding pocket. Exploitation of these differences enabled the development of an ATP-competitive inhibitor with approximately tenfold selectivity for PAK1 over PAK2 [15]. Although this degree of separation remains relatively modest, it indicates that local conformational preferences may be used to improve isoform discrimination even when the residues forming the ATP-binding site are highly conserved.

PAK1 also contains ligand-binding regions outside the conserved ATP cleft. Structural characterization of the dibenzodiazepine series that produced the reference allosteric inhibitor NVS-PAK1-1 identified a pocket adjacent to the ATP-binding site that becomes accessible in an inactive conformation of the kinase. Binding within this region stabilizes a DFG-out inactive conformation and indirectly prevents productive ATP binding without relying on conventional hinge interactions [16]. Structural differences within this allosteric environment, including residues that differ between PAK1 and PAK2, are considered to provide a more favorable basis for isoform discrimination than the highly conserved ATP-binding site [16,17].

**Figure 3:**
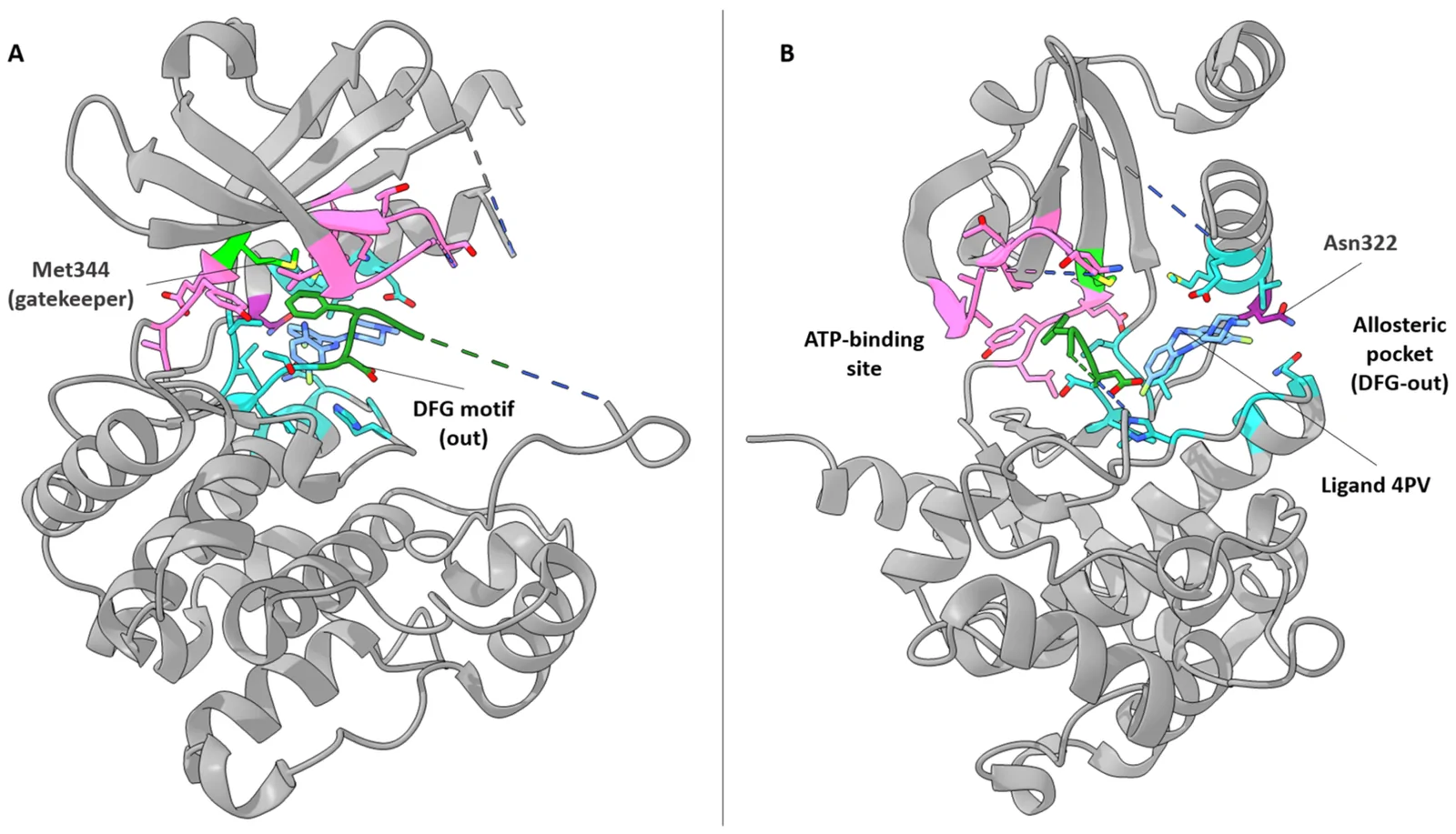
Structural features of the PAK1 kinase domain relevant to allosteric inhibition. (**A**) Standard kinase view of the PAK1 kinase domain in the inactive DFG-out conformation. The DFG motif is shown in dark green and the gatekeeper residue Met344 in light green. (**B**) Spatial relationship between the ATP-binding region and the adjacent allosteric pocket occupied by the dibenzodiazepine ligand 4PV in the PAK1 crystal structure (PDB ID: 4ZLO). The allosteric ligand is shown in light blue, residues defining the allosteric pocket in cyan and ATP-site residues in pink. Asn322, highlighted in purple, corresponds to Leu301 in PAK2 and contributes to the local structural environment associated with PAK1 selectivity. Dashed lines indicate chain discontinuities corresponding to residues unresolved in the original 4ZLO crystal structure.

The spatial relationship between the ATP-binding region and the adjacent allosteric pocket, together with selected structural features relevant to PAK1 selectivity, is illustrated in Figure 3 using a dibenzodiazepine precursor of NVS-PAK1-1, ligand 4PV (PDB ID: 4ZLO).

The conformation of the full-length protein may further influence the accessibility of both ATP-site and allosteric pockets. Recent PAK2 structures indicate that interactions between the regulatory and catalytic regions constrain the orientation of the kinase lobes and support an intramolecularly autoinhibited state [8]. Although these observations were obtained for PAK2, they suggest that differences in the conformational ensembles of group I PAKs may contribute to inhibitor selectivity beyond what can be predicted from catalytic-domain sequence comparisons alone.

An additional level of structural diversity arises from alternative splicing of the PAK1 transcript. In addition to the canonical full-length protein, a shorter variant lacking exon 15, generally referred to as PAK1Δ15, has been experimentally characterized [18]. Exon 15 exclusion alters the sequence of the C-terminal portion of the kinase domain and results in functional differences from full-length PAK1, including changes in intracellular localization and reduced activation of MAPK signaling in some experimental models [19]. However, the distribution and biological relevance of the two variants remain incompletely defined, and most pharmacological studies do not distinguish between them. It therefore remains unclear whether alternative splicing substantially affects inhibitor binding or cellular sensitivity to PAK1-directed compounds.

### 2.3. PAK1 Signaling and Disease Relevance

It is already established that PAK1 contributes to several signaling processes relevant to tumor progression, including cytoskeletal remodeling, migration, proliferation, survival and adaptation to therapeutic pressure. Its oncogenic effects arise through both catalytic and scaffold-related functions and involve interactions with MAPK, AKT, estrogen-receptor and adhesion-associated signaling networks [3,4,5]. However, PAK1 activity is unlikely to represent a universal dependency across tumor types, and increased expression or phosphorylation alone does not necessarily indicate that a tumor will respond to PAK1-directed treatment.

Recent evidence has strengthened the association between PAK1 activation and acquired treatment resistance. In estrogen receptor-positive breast cancer models resistant to combined fulvestrant and abemaciclib, increased PAK1 expression and phosphorylation were associated with enhanced proliferation and anchorage-independent growth as well as invasion. Genetic depletion or pharmacological inhibition of PAK1 reduced these phenotypes and restored sensitivity to endocrine therapy and CDK4/6 inhibition in cellular and xenograft models [20]. More recent phosphoproteomic analyses have also linked increased PAK1 activity to resistance to tamoxifen and long-term estrogen deprivation, although the downstream responses differ according to the form of endocrine pressure applied [21].

PAK1 may therefore be particularly relevant in tumors in which its activity forms part of an adaptive signaling state rather than acting as an isolated oncogenic driver. This interpretation is supported by studies reporting improved responses when PAK inhibition is combined with other therapeutic interventions, including Aurora A inhibition, PARP inhibition, or simultaneous modulation of other PAK family members [22,23,24]. These findings provide a rationale for investigating PAK1 as a sensitizing or resistance-associated target, while also emphasizing the need to identify molecular contexts in which its inhibition produces a measurable therapeutic advantage.

### 2.4. Pharmacological Challenges in Targeting PAK1

Potent inhibition of PAK1 has already been achieved with several chemical classes, yet converting biochemical activity into selective and therapeutically useful pharmacology remains difficult. The most evident obstacle remains the close relationship between the catalytic domains of PAK1, PAK2 and PAK3. As discussed above, most residues involved in ATP recognition are conserved, and therefore, many ATP-competitive compounds behave as group I PAK inhibitors rather than as truly PAK1-selective molecules. The recent structure-guided exploitation of local PAK1-PAK2 differences produced an approximately tenfold separation between the two isoforms, which represents meaningful progress, but may still be insufficient to ensure selective inhibition across the full range of concentrations reached in vivo [15].

Separation of PAK2 is particularly relevant because the two kinases do not possess equivalent physiological roles. PAK2 is widely expressed, and experimental deletion in endothelial cells impaired vascular development and adult blood-vessel maintenance [25]. Chemically distinct group I PAK inhibitors have also produced acute cardiovascular toxicity within relatively narrow exposure windows in preclinical studies, although these findings cannot be attributed exclusively to PAK2 inhibition, as the compounds differed in their broader kinome profiles [26]. The association between PAK2 and cardiovascular function implies, nevertheless, that modest biochemical selectivity may not translate into sufficient therapeutic margin, especially when sustained systemic exposure is required. For this reason, avoidance of PAK2 inhibition remains a major design objective in recent PAK1 research programs [15].

Selectivity across purified kinase panels is only part of the problem, as intracellular ATP concentrations, protein conformation, localization and the abundance of individual PAK isoforms can substantially alter inhibitor activity in cells. A compound with an attractive biochemical profile may therefore show weaker target engagement, additional cellular targets, or a phenotype appearing only at concentrations exceeding those needed for PAK1 inhibition. This issue is not unique to PAK1, but recent chemical proteomic and live-cell target-engagement studies have shown that kinase selectivity measured in cell-free assays may differ considerably from the interaction profile observed in intact cells [27,28]. Thus, for PAK1-directed studies, cellular target engagement and a proximal pharmacodynamic readout are more informative than antiproliferative activity alone.

Allosteric inhibition can reduce some of the discussed limitations associated with ATP-site conservation, although it introduces different constraints. NVS-PAK1-1 achieved substantial PAK1-over-PAK2 and broader kinome selectivity by exploiting an inactive conformation outside the conventional hinge-binding region [16]. However, its modest cellular effects in several models showed that biochemical selectivity does not necessarily produce a correspondingly strong phenotype. Conversion of the same PAK1-binding scaffold into the degrader BJG-05-039 improved antiproliferative activity in PAK1-dependent cells and selectively depleted PAK1 over PAK2, but also introduced the pharmacological requirements specific to targeted degradation, including the formation of a productive ternary complex and the control of proteome-wide degradation selectivity [17].

A further limitation arises from the organization of PAK1 signaling itself, as catalytic inhibition does not remove the protein from signaling complexes and may leave kinase-independent or scaffold-associated functions intact. Conversely, complete depletion may affect functions that are not involved in the disease phenotype under investigation. The relevant form of PAK1 modulation may therefore vary between models, and neither inhibition nor degradation can be assumed to reproduce the effects of genetic knockout without direct experimental comparison. Moreover, alternative splice forms, variable activation states and differences in subcellular recruitment add further sources of heterogeneity that are rarely examined in pharmacological studies.

Lastly, another problem remains that markers used to demonstrate PAK1 inhibition are inconsistent. Activation-loop phosphorylation is commonly measured, but phospho-PAK1 antibodies can also detect the homologous PAK2 site, and changes in downstream substrates can reflect the activity of several kinases. Recently developed live-cell assays for PAK isoform engagement and conformational state may help separate inhibitor binding from indirect pathway effects. Notably, current NanoBRET-based approaches can assess engagement of individual PAK isoforms, while newer assays suggest that group I inhibitors may themselves influence PAK dimerization in living cells [29,30]. These tools are still new, but they address a longstanding weakness of the field, as many compounds have been evaluated through phenotypic responses without equally strong evidence that PAK1 was selectively engaged under the same conditions. This issue is particularly relevant for commonly used tools such as PF-3758309, FRAX597, G-5555 and IPA-3, whose cellular phenotypes may reflect inhibition of multiple PAK isoforms or additional targets than PAK1 alone.

## 3. Pharmacological Approaches to PAK1 Modulation

Since its discovery as a target, PAK1 has been approached through several strategies that differ not only in their binding mode, but also in which functions of the protein they are expected to interfere with. ATP-competitive inhibition remains the most extensively studied approach, although the high conservation of the catalytic site has limited isoform selectivity, as already discussed. Allosteric compounds exploit regulatory conformation outside the conventional hinge-binding region, while targeted degradation aims to remove the entire PAK1 protein and therefore potentially affects both catalytic and non-catalytic functions. More recently, polypharmacological, combination-based and indirect strategies have broadened the concept of PAK1 modulation beyond selective inhibition of an isolated kinase target. Representative compounds from these approaches are summarized at the end of this section, in Table 1.

### 3.1. ATP-Competitive Inhibition

ATP-competitive inhibitors account for most of the early medicinal chemistry discoveries directed against PAK kinases. The availability of a well-defined catalytic pocket allowed substantial gains in biochemical potency, but selectivity has remained a recurrent limitation due to highly conserved residues in the ATP-binding site of group I PAKs. As a result, many compounds commonly used to investigate PAK1 biology are more accurately considered group I PAK inhibitors, while others possess additional kinase activities that complicate the interpretation of cellular phenotypes [4,40]. Accordingly, phenotypes observed with non-selective PAK inhibitors should not be interpreted as evidence of PAK1-specific function unless supported by isoform-selective pharmacology, genetic validation or direct cellular target-engagement data. Throughout this review, effects obtained with group I or broader PAK inhibitors are therefore distinguished from those that can be more confidently attributed to PAK1.

One of the earliest compounds to reach clinical development was PF-3758309, a potent ATP-competitive inhibitor originally developed against PAK4, but subsequently shown to inhibit all PAK family members together with several unrelated kinases [31]. The compound displayed strong antiproliferative and antitumor activity across multiple preclinical cancer models. However, because the compound inhibits several PAK isoforms and unrelated kinases, these phenotypes cannot be assigned specifically to PAK1. Clinical development was discontinued during phase I evaluation because of unfavorable pharmacokinetic behavior and limited evidence of antitumor efficacy as well as tolerability concerns [4,31]. Although PF-3758309 cannot be considered a selective PAK1 inhibitor, its development is still considered relevant as it illustrates the difference between broad preclinical activity and clinically useful PAK pharmacology.

A more informative group I series emerged from the FRAX compounds. FRAX597 is a potent group I PAK inhibitor and has been widely used in cellular and animal models, including NF2-associated schwannoma [32]. In these settings, the observed phenotypes are most appropriately interpreted as consequences of group I PAK inhibition rather than selective PAK1 blockade, unless supported by complementary isoform-specific experiments. Further optimization produced FRAX1036 and eventually G-5555, for which medicinal chemistry efforts improved permeability and kinase selectivity as well as hERG liability, while retaining potent group I PAK inhibition [33,41]. G-5555 showed a relatively restricted off-target kinase profile and inhibited tumor growth in PAK1-amplified breast cancer and non-small-cell lung cancer models. However, because G-5555 inhibits PAK1, PAK2 and PAK3 with high potency, its antitumor and toxicity phenotypes reflect group I PAK pharmacology rather than isolated PAK1 inhibition. As such, G-5555-related compounds produced acute cardiovascular toxicity at exposures close to those required for efficacy, substantially limiting their translational potential [26,33]. These findings reinforced the concerns that concurrent PAK2 inhibition may restrict the therapeutic window of ATP-site-binding PAK1 inhibitors.

Other medicinal chemistry studies have demonstrated that substantial kinome selectivity can be obtained without achieving true isoform selectivity within group I PAKs. For example, the development of bis-anilino pyrimidines by AstraZeneca resulted in compounds such as AZ13705339, which displayed subnanomolar PAK1 potency and relatively limited activity against unrelated kinases, while related analogues were optimized for improved pharmacokinetic properties [34]. These compounds have become valuable probes for group I PAK biology, but the similarity of PAK1, PAK2 and PAK3 catalytic sites continued to constrain isoform attribution. However, recent structure-guided work has provided evidence that this limitation is not absolute. Comparison of PAK1 with the first inhibitor-bound PAK2 crystal structure revealed differences in the conformation of the glycine-rich loop which could be exploited during ligand optimization [15]. The resulting ATP-competitive inhibitor, compound 35, achieved approximately tenfold selectivity for PAK1 over PAK2, despite the extensive conservation of the two catalytic domains [15]. Rather than solving the selectivity problem, this study demonstrates that local conformational preferences and induced-fit effects can provide additional design opportunities that are not evident from sequence comparison alone.

As such, ATP-site inhibition remains pharmacologically relevant, especially because potency, cellular permeability and exposure can be optimized using established kinase medicinal chemistry principles. Nevertheless, its principal limitation for PAK1 remains the need to separate target engagement from inhibition of closely related paralogs and other kinases. For this reason, ATP-competitive programs are most informative when biochemical profiling is accompanied by cellular target-engagement measurements and direct comparison against PAK2, rather than PAK1 alone.

### 3.2. Allosteric Inhibition

Allosteric inhibition emerged as an attractive alternative to ATP-site targeting because it offers access to less conserved regulatory regions of PAK1 and, therefore, a better prospect for isoform discrimination. One of the first compounds to validate this concept was IPA-3, identified through a screening study specifically to interfere with the autoregulatory mechanism of group I PAKs [35]. Rather than competing with ATP, IPA-3 binds the regulatory region and prevents activation of inactive PAK1 by Cdc42 or Rac1. Preactivated PAK1 is comparatively resistant to the compound, indicating that its activity depends on the conformational state of the kinase [35]. This mechanism provided an early proof that PAK1 could be inhibited outside the catalytic cleft, although the chemical properties of IPA-3 have limited its development beyond use as an experimental tool. Subsequent studies showed that IPA-3 covalently interacts with the regulatory domain and that its activity is strongly influenced by the cellular redox environment [36]. Reduction of the compound reverses its inhibitory activity, while nonspecific oxidative effects complicate interpretation at higher concentrations. Later, liposomal formulations have been explored in an attempt to improve its stability and in vivo exposure, with inhibition of prostate tumor growth reported in xenograft models, but these approaches have not overcome the intrinsic limitations of the scaffold [37]. Although IPA-3 was initially developed around the PAK1 autoregulatory mechanism, it also inhibits activation of related group I isoforms. Cellular effects obtained with IPA-3 should therefore generally be interpreted as evidence of group I PAK involvement unless PAK1-specific attribution is supported independently [35]. As such, IPA-3 remains mechanistically informative, particularly for distinguishing activation-dependent from ATP-site inhibition, but is poorly suited as a template for conventional drug development.

A more drug-like allosteric inhibition strategy arose from the dibenzodiazepine series developed by Novartis, culminating with NVS-PAK1-1 [16]. As discussed previously from a structural perspective, this compound class binds to a pocket adjacent to the ATP-binding site and stabilizes an inactive DFG-out conformation. More importantly, from a pharmacological point of view, NVS-PAK1-1 displays marked selectivity for PAK1 over PAK2, as well as a highly restricted kinome profile, providing one of the clearest demonstrations that PAK1 isoform selectivity can be achieved by moving away from conventional hinge-binding interactions [16]. This selectivity made the candidate a useful reference probe for further investigations of PAK1-specific biology, but was not without limitations, which were equally informative. Despite strong biochemical inhibition, NVS-PAK1-1 produced relatively modest downstream signaling changes and antiproliferative effects in several cellular models [4,17]. Whether this reflects insufficient cellular exposure, conformational dependence, redundancy with PAK2 and PAK3 or persistence of non-catalytic PAK1 functions varies between experimental systems. Nevertheless, the compound provided the chemical basis for subsequent PAK1-selective degraders, discussed further along, illustrating how an allosteric ligand with limited phenotypic potency can remain highly valuable as a selective recognition element [17].

Other non-ATP-site chemotypes have also been reported, including 1,4-naphthohydroquinone derivatives targeting the regulatory p21-binding region and showing preferential activity toward PAK1 and PAK3 over PAK2 [42]. Although these compounds expanded the range of possible allosteric binding modes, their micromolar potency and limited pharmacological characterization restricted their use compared to IPA-3 and NVS-PAK1-1. More generally, computationally predicted allosteric PAK1 inhibitors have appeared in the recent literature, but without matched structural validation, isoform profiling and cellular target-engagement data, such compounds should be distinguished from experimentally established allosteric probes.

Overall, allosteric inhibition has been more successful in demonstrating that PAK1 selectivity is achievable than in producing a clinically advanced inhibitor. Its main advantage lies in exploiting regulatory conformations that substantially differ between closely related PAK isoforms, while its principal limitation remains the dependence of inhibitor activity on protein conformation and activation state. These features make allosteric ligands particularly useful both as mechanistic probes and as starting points for alternative pharmacological modalities.

### 3.3. Targeted Degradation

One of the more substantial recent changes in PAK1 pharmacology came not from the identification of another inhibitory site, but from using an already selective PAK1 ligand as the recognition element of a proteolysis-targeting chimera (PROTAC). The first reported PAK1-selective degrader, BJG-05-039, was obtained by linking NVS-PAK1-1 to lenalidomide, thereby recruiting the cereblon (CRBN) E3 ubiquitin ligase and directing PAK1 toward proteasome-dependent degradation [17]. In this setting, the selectivity of the allosteric warhead could be retained, while the pharmacological outcome changed from occupancy-driven inhibition to removal of the target protein.

BJG-05-039 induced dose- and time-dependent degradation of PAK1 in MCF7 and OVCAR3 cells while largely sparing PAK2, with maximal degradation observed at nanomolar concentrations [17]. Degradation was prevented by disruption of CRBN engagement or proteasome activity, confirming that the effect depends on formation of the expected degradation machinery. Interestingly, higher concentrations reduced rather than increased PAK1 depletion, consistent with the characteristic PROTAC “hook effect”, in which excessive binary binding prevents the formation of a productive ternary complex. The compound also reduced MEK1 Ser298 and downstream ERK phosphorylation at concentrations where the parent molecule NVS-PAK1-1 produced little detectable effect, and its antiproliferative activity was markedly enhanced in PAK1-dependent cell lines [17]. The study is particularly relevant as it provides a direct comparison between selective inhibition and selective degradation using closely related chemical recognition of the same target. In the tested cells, BJG-05-039 produced antiproliferative effects in the low-nanomolar range, whereas micromolar concentrations of NVS-PAK1-1 were required to achieve comparable effects. Additionally, this improvement was not reproduced in cell lines preferentially dependent on PAK2 [17]. These observations support the idea that degradation can amplify the pharmacological consequences of an otherwise modest PAK1 inhibitor, although they do not establish that degradation will invariably outperform catalytic inhibition. Differences in protein turnover, E3-ligase expression, cellular permeability and dependence on non-catalytic PAK1 functions are likely to influence the response in each biological context.

A subsequent series expanded this proof of concept by designing both VHL- and CRBN-recruiting PAK1 degraders and identified compound **19s** as the most promising candidate [38]. In MDA-MB-231 triple-negative breast cancer cells, **19s** induced detectable PAK1 degradation from 0.1 μM and inhibited proliferation with an IC_50_ of approximately 1.27 μM. The compound also reduced cellular migration and produced tumor regression in an in vivo breast cancer model [38]. Unlike the initial BJG-05-039 study, focused primarily on establishing selective degradation and mechanistic validation, this work provided an early indication that PAK1 degradation can also produce measurable antitumor activity in vivo.

Despite these encouraging results, targeted degradation introduces its own limitations. Degrader efficacy depends on the geometry and cooperativity of the ternary complex formed between PAK1, the PROTAC and the recruited E3 ligase, meaning that the linker composition and ligase choice can be as important as the affinity of the PAK1-binding warhead itself. Their comparatively high molecular weight may also compromise permeability and pharmacokinetic behavior, while proteome-wide degradation profiles are required to distinguish PAK1 selectivity from unintended degradation mediated by the E3-ligase recruiter. In addition, the available evidence is still based on a small number of degrader series and preclinical models. Targeted degradation should therefore be considered an emerging complementary strategy rather than an established replacement for conventional PAK1 inhibition. Still, this modality has added an important experimental dimension to the field. By eliminating the PAK1 protein rather than solely blocking the transfer of the phosphate group, degraders offer a way to investigate whether the phenotypes attributed to PAK1 depend on catalytic activity, scaffolding functions, or both. For this reason, BJG-05-039 and compound **19s** represent particularly relevant additions to the current PAK1 pharmacological toolkit.

### 3.4. Polypharmacology and Combination-Based Therapies

Because PAK1 operates within interconnected signaling networks rather than as an isolated oncogenic driver, simultaneous modulation of complementary targets has been increasingly explored as an alternative to highly selective single-agent inhibition. This can be achieved either within the same molecule, through deliberate polypharmacology, or by combining a PAK1-directed compound with another therapeutic agent. The two strategies address a similar problem, that is, the capacity of tumor cells to maintain signaling through parallel or compensatory pathways, but differ considerably in their pharmacological flexibility and interpretability.

An interesting recent example of deliberate polypharmacology is ZMF-25, developed as a combined inhibitor of PAK1 and the class IIb histone deacetylases HDAC6 and HDAC10 [39]. The compound inhibited PAK1, HDAC6 and HDAC10 with IC_50_ values of 33, 64 and 41 nM, respectively, while maintaining selectivity over other tested PAK and HDAC isoforms. In MDA-MB-231 triple-negative breast cancer cells, ZMF-25 inhibited proliferation and migration, promoted mitochondrial dysfunction and reactive oxygen species accumulation and induced autophagy-associated cell death through modulation of AKT-mTOR signaling [39]. Antitumor activity was also reported in vivo together with favorable pharmacokinetic properties and no evident short-term toxicity. Rather than attempting to isolate the contribution of PAK1, this design deliberately combines kinase and epigenetic modulation within a single molecule, making ZMF-25 a useful example of how the pharmacological objective can shift from isoform purity toward coordinated pathway interference.

Combination therapy preserves greater control over the contribution and dosing of the individual components. A recent study in triple-negative breast cancer used the selective allosteric inhibitor NVS-PAK1-1 together with established cytotoxic drugs and reported synergistic effects with doxorubicin, paclitaxel and methotrexate [43]. Combined treatment reduced colony formation, migration and invasion at lower effective chemotherapy concentrations, while enhanced antitumor activity and suppression of metastasis were reproduced in orthotopic mammary tumor models [43]. These results are particularly relevant because NVS-PAK1-1 alone has shown comparatively modest phenotypic activity in several cellular systems, and its value may therefore become more apparent when PAK1 inhibition interferes with adaptive responses elicited by another treatment.

Other recent findings extend this rationale beyond conventional chemotherapy. In high-grade serous ovarian cancer models, PAK inhibition increased the susceptibility of tumor cells to cytotoxic CD8+ T-cell-mediated killing, while addition of PD-1 blockade further enhanced the effect [44]. Because the pharmacological inhibitors used in this study were not PAK1-selective, the results cannot be attributed to PAK1 alone. However, they introduce an additional context in which suppression of PAK signaling may influence tumor-cell-intrinsic proliferation or invasion, as well as the interaction between malignant cells and antitumor immunity.

PAK1 activity may also function as a marker for selecting therapies that do not directly inhibit PAK1. A longitudinal phosphoproteomic analysis of recurrent colorectal liver metastases identified increased PAK activity in tumors with poor response to chemotherapy and subsequently correlated PAK1 kinase activity with drug-sensitivity profiles across colorectal cancer cell lines [45]. PI3K inhibitors emerged prominently from this analysis, and copanlisib suppressed growth of 5-fluorouracil-resistant colorectal cancer models with high PAK1 expression, both in vitro and in vivo, while reducing PI3K-PAK1 pathway activity [45]. In this setting, PAK1 serves less as the immediate pharmacological target and more as a measurable signaling state that exposes an upstream therapeutic vulnerability.

These approaches illustrate why polypharmacology and combinations should not be considered merely secondary solutions for an insufficiently selective target. In some disease contexts, simultaneous perturbation of PAK1 and a cooperating pathway may be mechanistically preferable to complete dependence on PAK1 inhibition only. However, as the number of pharmacologically affected targets increases, attribution of efficacy and toxicity becomes more difficult. The value of these strategies will therefore depend on whether the selected combinations are supported by a defined biological relationship rather than by additive cytotoxicity.

### 3.5. Emerging Indirect and Non-Canonical Approaches

Direct occupancy of the catalytic or allosteric sites is not the only way in which PAK1 signaling can be altered. Because activation and localization of the kinase depend on a network of adaptor, scaffold and exchange proteins, interference with these interactions may offer a more indirect means of reducing PAK1 activity. This concept has recently gained renewed attention, particularly as the limitations of complete kinase inhibition and the importance of spatially organized PAK1 signaling have become more evident [46].

Several accessory proteins participate in the recruitment and activation of PAK1, including NCK1, GRB2, PIX-family exchange factors, GIT proteins and paxillin. Their interactions position PAK1 at membrane compartments and focal adhesions where Rac1/Cdc42 activation and downstream substrate phosphorylation occur [2,46]. Conversely, proteins such as merlin, nischarin, CRIPAK and PAK1IP1 can restrain PAK1 activation or localization [46]. Pharmacologically altering these regulatory interactions could therefore reduce pathological PAK1 signaling without necessarily abolishing all functions of the kinase. At the moment, however, most of these proteins remain mechanistic rather than validated therapeutic targets.

Disruption of PAK1 protein–protein interactions has already provided experimental proof of principle. Peptides derived from the NCK- or PIX-binding regions of PAK1 can interfere with its intracellular recruitment, and TAT-pak18, which contains the PIX-interacting motif, was reported to preferentially suppress growth of ovarian cancer cells with activated PAK1 [4,47]. Similar peptide strategies targeting the PAK-NCK interaction have altered endothelial migration and contractility [4]. Their therapeutic development has been limited mainly by delivery, stability and pharmacokinetic constraints, but they demonstrate that PAK1 signaling can be disrupted without directly occupying its kinase domain.

Targeting the accessory protein itself represents another possibility. The interaction between NCK1 and PAK1 is required for the recruitment of PAK1 to activated receptor complexes, while PIX/GIT complexes contribute to focal-adhesion localization and local Rac signaling [46]. Small-molecule inhibition of such adaptor interactions has been achieved in other signaling settings, as is the case of AX-024, which binds the SH3 domains of NCK proteins and disrupts selected NCK-mediated protein interactions, although it has not yet been developed specifically as a PAK1-directed agent [46]. Accordingly, the current evidence supports the druggability of the regulatory network more strongly than it supports any particular accessory protein as an established PAK1 therapeutic agent.

Another viable approach, even less direct, is represented by the upstream pathway modulation strategy. Since Rac1 and Cdc42 are major regulators of group I PAK activation, inhibition of their activation, membrane localization or guanine-nucleotide exchange can reduce PAK signaling downstream. The limitation, however, is specificity, as both GTPases regulate numerous effectors besides PAK1, making it difficult to attribute biological effects to PAK suppression alone. A similar problem applies to targeting receptor or PI3K-dependent inputs that influence PAK1 activation. Such approaches may be relevant when PAK1 functions as part of a defined signaling dependency rather than as the principal pharmacological target, as illustrated by the recently described PI3K-PAK1 vulnerability in recurrent colorectal liver metastases [45].

Emerging work on regulatory RNAs adds another layer to this concept. Several microRNAs and long non-coding RNAs have been reported to influence PAK1 expression or upstream Rac1/Cdc42 signaling, including lncRNA-dependent regulation of the Cdc42-PAK1 axis in cancer models [46]. Currently, these findings remain primarily mechanistic and lack the pharmacological validation available for direct inhibitors or degraders. Nevertheless, they broaden the range of interventions through which aberrant PAK1 signaling might eventually be modulated.

Indirect approaches remain considerably less mature than ATP-competitive inhibition, allosteric modulation or targeted degradation. However, their potential advantage is that they may interfere selectively with a disease-relevant component of PAK1 signaling, such as recruitment to a particular signaling complex, while preserving functions performed elsewhere in the cell. Whether this can translate into improved efficacy or safety is largely unresolved, and the field currently lacks well-characterized small molecules that selectively modulate a PAK1-specific protein interaction in vivo.

## 4. Recent Findings

The recent development of more selective inhibitors and new pharmacological modalities has renewed the question of how extensively PAK1-active compounds have actually been characterized against the closely related PAK2 and PAK3 isoforms. In order to obtain a broader view of the available pharmacological landscape, we examined the current ChEMBL bioactivity datasets for the three human group I PAKs. ChEMBL contained 2769 bioactivity records for PAK1, 2075 for PAK2 and 1621 for PAK3, corresponding to 2039, 1314 and 948 unique compounds, respectively. Although 729 molecules were represented across all three datasets, only 102 of these compounds had quantitative IC_50_, K_i_ or Kd measurements available for all three isoforms. Thus, the substantial overall overlap between the datasets largely overestimates the number of compounds for which direct quantitative comparison across group I is possible. Among PAK1 records with an assigned publication year, 341 bioactivity records corresponding to 284 unique compounds were associated with documents published between 2021 and 2025, indicating that substantial pharmacological characterization of PAK1 has continued in recent years.

For a more focused comparison, compounds with at least one standardized PAK1 IC_50_, K_i_ or Kd value below 100 nM were considered a potent PAK1 subset, resulting in 154 unique molecules. Compounds were matched between the three datasets by their ChEMBL molecule identifiers, and homolog profiling was assessed using available standardized IC_50_, K_i_ or K_d_ measurements. Absence of an activity record against PAK2 or PAK3 was considered missing information rather than evidence of PAK1 selectivity. The underlying compound-level dataset used for this analysis is provided in the Supplementary Materials (Table S1). The resulting distribution is summarized in Table 2.

The comparison reveals a substantial gap between the amount of potency information available for PAK1 and the amount of data that can actually be used to evaluate isoform selectivity. Of the 154 potent PAK1 compounds, only 25 (16.2%) had any PAK2 bioactivity information and only 17 (11.0%) had corresponding data for PAK3. Quantitative IC_50_/K_i_/Kd measurements were available for even fewer compounds, 22 against PAK2 and 12 against PAK3. For 129 of the 154 compounds (83.8%), neither PAK2 nor PAK3 bioactivity records were available, meaning that isoform selectivity cannot be assessed from the current ChEMBL dataset. Notably, all 17 potent PAK1 compounds for which PAK3 data were available were also represented in the PAK2 dataset, indicating that PAK3 characterization within this subset largely occurred as part of broader group I PAK profiling.

Where quantitative homolog measurements were available, cross-isoform activity was common rather than exceptional. This subset is likely enriched for compounds subjected to broader homolog or kinase-panel profiling and should therefore not be considered representative of the entire PAK1 dataset. Twenty-one of the 22 potent PAK1 compounds quantitatively characterized against PAK2 also showed PAK2 potency below 1 μM, while 11 of 12 compounds evaluated against PAK3 met the same threshold. Fifteen compounds retained sub-100 nM potency against PAK2 and eight against PAK3. These observations are consistent with the high structural similarity of the group I catalytic domains, but they should not be interpreted as indicating that the same proportions apply to all 154 PAK1-active compounds. Compounds subjected to detailed homolog profiling are unlikely to represent a random sample and may be enriched for molecules already suspected to show broader group I PAK activity.

The main conclusion from this database snapshot is, therefore, not that most potent PAK1 inhibitors necessarily lack selectivity, but rather that selectivity cannot currently be established for most of the compounds from publicly available homologous bioactivity data. This distinction is important, as a compound reported only against PAK1 should be considered insufficiently characterized, not PAK1-selective. Conversely, the high frequency of submicromolar PAK2 and PAK3 activity among compounds for which matched measurements do exist reinforces the value of systematic paralog profiling when new PAK1 inhibitors are reported. In this respect, recent structure-guided studies, cellular target-engagement methods and broader kinome profiling represent an important change in how PAK1 pharmacology can be evaluated, moving the field from demonstration of potency toward more rigorous assessment of isoform attribution.

## 5. Critical Discussion and Future Directions

The current PAK1 pharmacological landscape presents an apparent contradiction. A considerable number of chemically diverse compounds can inhibit PAK1 with high biochemical potency, yet no PAK1-selective therapeutic agent has reached clinical use. The main limitation is therefore no longer simple target druggability, but the ability to connect biochemical potency with isoform selectivity, cellular target engagement and a disease context in which modulation of PAK1 provides a sufficient therapeutic advantage. The ChEMBL snapshot presented above reflects the same imbalance from another perspective: potent PAK1-associated activity measurements are numerous, whereas matched information allowing direct comparison with PAK2 is available for only a small fraction of these compounds.

Several pharmacological approaches may address different components of this problem, but none currently provides a complete solution. Further exploitation of local conformational differences within the ATP-binding region may improve PAK1/PAK2 discrimination, as recent structure-guided work has already demonstrated [15]. Allosteric inhibitors provide another route toward selectivity by accessing less conserved conformational states, whereas targeted degradation may be advantageous when removal of scaffold or other non-catalytic functions contributes to the desired phenotype [16,17,38]. These approaches should not necessarily be regarded as successive generations in which one replaces another. They can develop independently, and their relative value is likely to differ according to tissue exposure, tumor dependency, activation state and the contribution of catalytic versus non-catalytic PAK1 functions.

Improved pharmacological tools will also require more informative experimental validation. Direct cellular target-engagement assays, broader kinome profiling and paired measurement of PAK1 and PAK2 activity should increasingly accompany conventional biochemical potency data. Full-length structural information may become equally important, since differences in regulatory-domain interactions and conformational dynamics are not captured by comparison of isolated kinase domains alone [8]. The biological side of the problem requires similar refinement. Rather than selecting models only on the basis of high PAK1 expression or phosphorylation, future studies would benefit from identifying molecular or phosphoproteomic signatures that indicate a genuine dependence on PAK1 signaling. Recent findings in therapy-resistant breast cancer and recurrent colorectal metastases suggest that such dependencies may emerge particularly during adaptive responses to treatment [20,21,45].

Combination therapy and deliberate polypharmacology represent another route that should develop in parallel. In tumors where PAK1 participates in a broader resistance network, complete selectivity for PAK1 may not necessarily provide the greatest therapeutic effect. The relevant challenge is to distinguish rational pathway co-dependencies from nonspecific combinations that merely increase cytotoxicity. In this respect, longitudinal phosphoproteomics, functional genetic screening and treatment-response profiling could be particularly useful for identifying situations in which PAK1 modulation is mechanistically justified.

Taken together, these observations indicate that future PAK1 drug-discovery efforts will need to address several complementary priorities rather than a single limiting factor. Improving isoform discrimination, strengthening cellular target engagement validation, expanding allosteric and degradation-based approaches and identifying disease contexts that genuinely depend on PAK1 signaling are likely to progress in parallel. At the same time, rational combination strategies and more systematic generation of matched PAK1/PAK2 pharmacological data may improve both compound development and interpretation of biological effects. The main directions emerging from the current evidence are summarized in Table 3.

Several of these priorities may also benefit from the growing availability of kinase bioactivity, structural and omics datasets, which creates an opportunity for more data-driven approaches. Protein kinases are particularly suitable for this type of analysis because unusually large amounts of inhibitor activity and structural information are publicly available, and recent machine-learning approaches increasingly model kinase selectivity across multiple targets simultaneously rather than predicting affinity for one kinase in isolation [48]. For PAK1, models integrating PAK1 and PAK2 activity measurements with structural conformations, kinome-wide profiling and cellular response data could help prioritize chemotypes with a better predicted selectivity window or identify underexplored regions of chemical space. Similar approaches could potentially integrate transcriptomic or phosphoproteomic information to select disease contexts in which PAK1 dependence is more likely. AI/ML methods in kinase drug discovery already address multi-target selectivity, lead optimization and multi-objective properties such as potency, selectivity and pharmacokinetics, although their performance is currently strongly dependent on the quality and completeness of the underlying datasets [49].

For PAK1 specifically, that limitation is rather important, as the heterogeneous assay formats and extensive missing paralog measurements observed in current databases mean that an algorithm cannot compensate for absent experimental information. Data-driven predictions may therefore be most useful for prioritizing experiments rather than replacing them. Systematic generation of matched PAK1/PAK2 datasets, cellular engagement measurements and standardized phenotypic readouts would substantially increase the value of both conventional medicinal chemistry and future computational approaches.

At present, it remains difficult to predict which strategy will provide the fastest route toward clinically useful PAK1 modulation. Improved ATP-site selectivity, allosteric inhibition, targeted degradation and context-dependent combination approaches each address different limitations of the target and may continue to progress independently. Determining which of them offers the best balance between efficacy, selectivity and safety will require closer integration of medicinal chemistry, structural biology and disease-specific pharmacology.

## 6. Conclusions

PAK1 remains a pharmacologically attractive but challenging therapeutic target. Although potent inhibition has been achieved with several chemical classes, translation has been limited by the close similarity of group I PAKs, incomplete separation from PAK2, variable cellular target engagement and the contribution of non-catalytic PAK1 functions. The main challenge is therefore no longer biochemical inhibition itself, but achieving selective and biologically meaningful modulation in the appropriate disease context.

Recent structural studies, allosteric inhibitors, PAK1 degraders and combination-based strategies have expanded the possibilities for PAK1 modulation beyond conventional ATP-site inhibition. At the same time, available bioactivity data show that potent PAK1-associated compounds considerably outnumber those with convincing paralog-selectivity information. Future progress will likely depend on integrating medicinal chemistry with structural data, direct cellular target-engagement measurements and better identification of PAK1-dependent disease states. ATP-competitive inhibition, allosteric modulation, degradation and rational combination strategies can continue to develop in parallel, and it remains to be established which approach will provide the best balance between efficacy, selectivity and safety.

## Figures and Tables

**Table 1:** Representative compounds used for pharmacological modulation of PAK1.

Compound/Reference	Structure	Pharmacological Approach/ Binding Mode and Potency	Selectivity/ Relevant Target Profile	Representative Biological Significance
PF-3758309 [31]	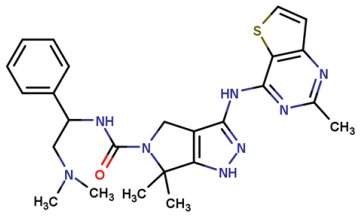	ATP-competitive; broad PAK inhibitor; PAK1 IC_50_ ≈ 13.7 nM	Broad activity across PAK1–PAK6; PAK2 190 nM; PAK3 99 nM; PAK4 18.7 nM; PAK5 18.1 nM; PAK6 17.1 nM; Additional off-target kinases	Broad preclinical antitumor activity; target attribution remains non-specific. Entered phase I but development was discontinued because of unfavorable pharmacokinetics, tolerability and limited efficacy
FRAX597 [32]	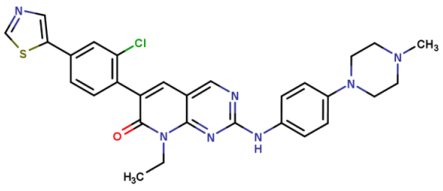	ATP-competitive; group I PAK inhibitor; PAK1 IC_50_ ≈ 7.7 nM	PAK2 12.8 nM; PAK3 19.3 nM; Weak activity toward group II PAKs	Widely used group I PAK probe; Antitumor activity in NF2-associated and cancer models; effects reflect group I PAK inhibition. Not PAK1-selective
G-5555 [33]	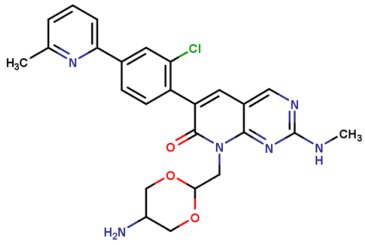	ATP-competitive; group I PAK inhibitor; PAK1 K_i_ ≈ 3.7 nM	PAK2 K_i_ ≈ 11 nM; Restricted broader kinome profile	Improved drug-like properties and in vivo group I PAK activity; Cardiovascular toxicity limited translational potential
AZ13705339 [34]	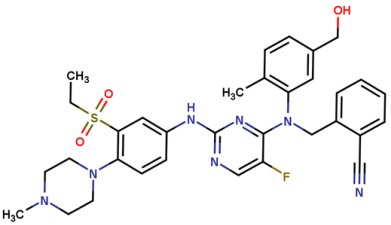	ATP-competitive; bis-anilino pyrimidine; PAK1 IC_50_ ≈ 0.33 nM	PAK2 ≈ 6 nM; >7500-fold over PAK4; Restricted kinome profile	Highly potent probe with cellular phosphorylated PAK1 inhibition; Still limited by incomplete PAK1/PAK2 separation
Compound 35, Johns et al. [15]	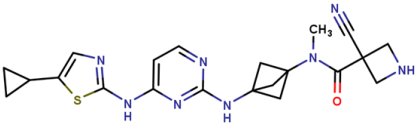	ATP-competitive; structure-guided inhibitor;	Approximately 10-fold PAK1-over-PAK2 selectivity	Exploits P-loop/glycine-rich-loop conformational differences; Recent proof that ATP-site isoform discrimination is achievable
IPA-3 [35,36,37]	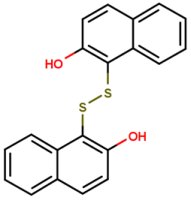	Allosteric; regulatory-domain inhibitor; PAK1 IC_50_ ≈ 2.5 μM	Preferential group I PAK inhibition; Activation-state dependent	Mechanistic group I PAK probe; Covalent/redox-sensitive behavior and limited stability restrict drug development
NVS-PAK1-1 [16]	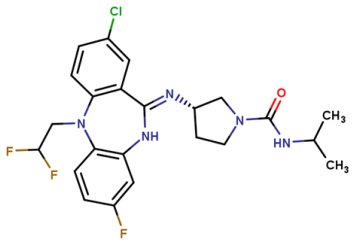	Allosteric; stabilizes inactive DFG-out conformation; PAK1 IC_50_ ≈ 5 nM	Marked PAK1-over-PAK2 selectivity; Highly restricted kinome profile	One of the most selective PAK1 probes; Modest cellular phenotype but valuable scaffold for degrader development
BJG-05-039 [17]	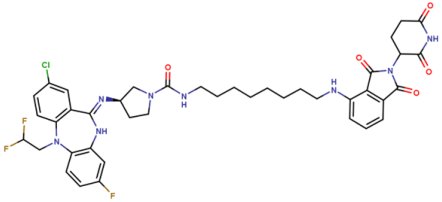	PROTAC; CRBN-recruiting PAK1 degrader	Selectively degrades PAK1 while largely sparing PAK2	Nanomolar degradation; Stronger antiproliferative effects than NVS-PAK1-1 in PAK1-dependent cells
Compound **19s**, Du et al. [38]	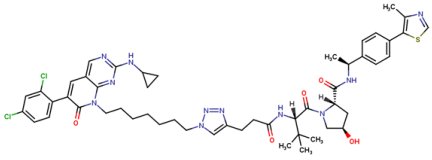	PROTAC; PAK1 degrader	Designed for selective PAK1 degradation	Reduced proliferation and migration in TNBC cells and showed in vivo antitumor activity; Further PK/selectivity validation needed
ZMF-25 [39]	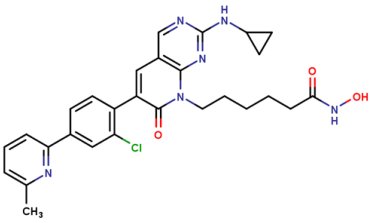	Polypharmacological PAK1/HDAC6/HDAC10 inhibitor; PAK1 IC_50_ ≈ 33 nM	HDAC6 64 nM; HDAC10 41 nM; Selectivity over other tested PAK/HDAC isoforms	Antiproliferative and antimigratory effects in TNBC with in vivo activity; Efficacy reflects deliberate multi-target inhibition

**Table 2:** ChEMBL snapshot analysis of PAK1, PAK2 and PAK3 bioactivities.

Analysis Level	Parameter	PAK1	PAK2	PAK3
Overall ChEMBL dataset	Bioactivity records	2769	2075	1621
	Unique compounds	2039	1314	948
Cross-isoform coverage	Compounds shared with PAK1	—	780	745
	Compounds represented across PAK1 PAK2 and PAK3	729	729	729
Potent PAK1 subset *	Potent PAK1 compounds	154	—	—
	Potent PAK1 compounds with any homolog bioactivity data	—	25 (16.2%)	17 (11.0%)
	Potent PAK1 compounds with quantitative homolog IC_50_/K_i_/K_d_ data	—	22 (14.3%)	12 (7.8%)
	Potent PAK1 compounds with homolog potency < 1 µM	—	21 (13.6%)	11 (7.1%)
	Potent PAK1 compounds with homolog potency < 100 nM	—	15 (9.7%)	8 (5.2%)
Among quantitatively profiled potent PAK1 compounds	Homolog potency < 1 µM	—	21/22 (95.5%)	11/12 (91.7%)

Data were retrieved from ChEMBL on 28–31 August 2026 for the human single-protein targets PAK1 (CHEMBL4600), PAK2 (CHEMBL4487) and PAK3 (CHEMBL2999). Compounds were matched by Molecule ChEMBL ID. * Potent PAK1 compounds were defined as unique molecules with at least one standardized IC_50_, K_i_ or Kd value < 100 nM. Quantitative homolog profiling used standardized IC_50_, K_i_ or Kd measurements with exact or upper-bound relations where the respective potency threshold could be established. Absence of homolog bioactivity data was treated as unknown selectivity.

**Table 3:** Future directions for PAK1 inhibitor design and pharmacological development.

Future Directions	Rationale	Key Considerations
Improving PAK1/PAK2 discrimination	ATP-site conservation remains the main obstacle to isoform-selective inhibition	Selectivity should be evaluated directly against PAK2 and, where possible, PAK3; modest biochemical separation may not translate into cellular selectivity
Developing allosteric inhibitors	Less conserved conformational pockets may offer better isoform selectivity	Binding can depend strongly on protein conformation and activation state, so structural validation and cellular engagement remain important
Expanding targeted degradation	Degradation can remove both catalytic and scaffold functions of PAK1	Activity depends on warhead affinity, linker geometry, E3-ligase recruitment, permeability and proteome-wide degradation selectivity
Strengthening cellular target-engagement studies	Biochemical potency does not ensure selective intracellular PAK1 inhibition	Intracellular ATP levels, protein conformation and isoform abundance may alter engagement relative to cell-free assays
Identifying PAK1-dependent disease contexts	High PAK1 expression or phosphorylation does not necessarily imply functional dependency	Model selection should rely on functional or molecular evidence of PAK1 dependence rather than expression alone
Developing rational combinations and polypharmacology	PAK1 often functions within adaptive signaling networks rather than as an isolated driver	Combination partners should reflect defined pathway co-dependencies, while efficacy and toxicity become harder to attribute as more targets are affected
Improving pharmacological datasets and data-driven prioritization	Matched paralog measurements remain limited, restricting both interpretation and computational modeling	Dataset completeness, assay consistency and matched PAK1/PAK2/PAK3 measurements are critical; computational predictions cannot compensate for missing experimental data

## Data Availability

No new data were created or analyzed in this study. Data sharing is not applicable to this article.

## Supplementary Materials

The following supporting information can be downloaded at https://www.mdpi.com/article/10.3390/ph19101628/s1, Table S1: ChEMBL dataset used for the comparative analysis of PAK1, PAK2 and PAK3 bioactivities.

## Abbreviations

The following abbreviations are used in this manuscript:

AI/ML	Artificial Intelligence/Machine Learning
AKT	Protein kinase B
ATP	Adenosine triphosphate
CDK4/6	Cyclin-dependent kinases 4 and 6
CK2	Casein kinase 2
CRBN	Cereblon
CRIB	Cdc42/Rac-interactive binding
CRIPAK	Cysteine-rich inhibitor of PAK1
DFG	Aspartate–phenylalanine–glycine motif
ERK	Extracellular signal-regulated kinase
GIT	G protein-coupled receptor kinase-interacting protein
GRB2	Growth factor receptor-bound protein 2
GTP	Guanosine triphosphate
HDAC	Histone deacetylase
HDAC6	Histone deacetylase 6
HDAC10	Histone deacetylase 10
hERG	Human ether-à-go-go-related gene potassium channel
IC_50_	Half-maximal inhibitory concentration
Kd	Equilibrium dissociation constant
K_i_	Inhibition constant
lncRNA	Long non-coding RNA
MAPK	Mitogen-activated protein kinase
MEK	Mitogen-activated protein kinase kinase
mTOR	Mechanistic target of rapamycin
NanoBRET	NanoLuc bioluminescence resonance energy transfer
NCK	Non-catalytic region of tyrosine kinase adaptor protein
NCK1	Non-catalytic region of tyrosine kinase adaptor protein 1
NF2	Neurofibromatosis type 2
NSCLC	Non-small-cell lung cancer
PAK	p21-activated kinase
PAK1IP1	PAK1-interacting protein 1
PARP	Poly(ADP-ribose) polymerase
PD-1	Programmed cell death protein 1
PI3K	Phosphoinositide 3-kinase
PIX	PAK-interacting exchange factor
PROTAC	Proteolysis-targeting chimera
SH3	Src homology 3
TNBC	Triple-negative breast cancer
VHL	Von Hippel–Lindau
